# Anterior Mitral Leaflet Flutter on M-mode Echocardiography as an Indicator of Atrial Fibrillation: Case Report

**DOI:** 10.5811/cpcem.39988

**Published:** 2025-05-01

**Authors:** Maya Alexandri, Adam Church, Chelsea Ausman, Dan Brillhart

**Affiliations:** * Carl R. Darnall Army Medical Center, Department of Emergency Medicine, Fort Cavazos, Texas; † Rhine Ordnance Barracks, Department of Resuscitative Surgery, Rhineland-Palatinate, Germany

**Keywords:** POCUS, point-of-care ultrasound, transthoracic echocardiogram, TTE, M-mode, aortic regurgitation, atrial fibrillation, anterior mitral leaflet flutter, case report

## Abstract

**Introduction:**

M-mode in bedside point-of-care ultrasound (POCUS) transthoracic echocardiography (TTE) remains an important tool for emergency physicians. M-mode of the mitral valve is used to assess ejection fraction (EF) during assessment of E-point septal separation (EPSS). Anterior mitral leaflet fluttering visualized on M-mode echocardiography is a known sequela of aortic regurgitation. Although not previously reported in the emergency medicine (EM) literature, anterior mitral leaflet fluttering also occurs with atrial fibrillation.

**Case Report:**

We present the first case in peer-reviewed EM literature of anterior mitral leaflet fluttering observed on M-mode echocardiography caused by atrial fibrillation. Our patient was a 54-year-old male who had a POCUS TTE that showed anterior mitral leaflet fluttering on EPSS. Subsequent inpatient workup confirmed the diagnosis of symptomatic atrial fibrillation without ischemia or clinically significant aortic regurgitation.

**Conclusion:**

Emergency physicians must rapidly assess and risk-stratify undifferentiated patients presenting with chest pain. Understanding that anterior mitral leaflet fluttering on M-mode during E-point septal separation may signal atrial fibrillation augments efficient and appropriate disposition of these patients.

## INTRODUCTION

Point-of-care ultrasound (POCUS) transthoracic echocardiography (TTE) is an important and frequently used tool for evaluation of the undifferentiated emergency department (ED) patient with chest pain, dyspnea, palpitations, or a dysrhythmia observed on electrocardiogram (ECG). In assessing ejection fraction (EF), emergency physicians may estimate EF visually. E-point septal separation (EPSS) ascertained using M-mode echocardiography is among the most accessible options for quantifying the EF and supporting the visual estimate.

M-mode echocardiography depicts motion by isolating a single raster line of two-dimensional (2D) echocardiography.^1^ While 2D and Doppler ultrasound have overshadowed M-mode in past decades, M-mode remains valuable in a range of contexts, especially where temporal resolution and a high frame rate are necessary for detecting pathology.^2^ These qualities carry special weight in assessment of the mitral valve, which is likely “the fastest moving structure within the heart.”^2^ M-mode facilitates assessment of certain aberrant mitral valve motion, including “the fine fluttering associated with aortic regurgitation.”^1^, ^2^

“[F]ine fluttering” of the mitral valve also appears to be a manifestation of atrial fibrillation.^3^, ^4^ Based on our literature search, this association with atrial fibrillation is not widely known or discussed in the emergency medicine (EM) literature. We present the first case in peer-reviewed EM literature of anterior mitral leaflet fluttering observed on M-mode echocardiography during EPSS assessment, favored to represent a sequela of the patient’s underlying atrial fibrillation.

## CASE REPORT

Our patient was a 54-year-old male, active-duty soldier with a past medical history of paroxysmal atrial fibrillation (onset in the prior year, anti-coagulated on rivaroxaban, rate-controlled on diltiazem, and adherent to his medications), hypertension, and obstructive sleep apnea, presenting with chest pain, shortness of breath, and palpitations that woke him from sleep. He described the chest pain as left-sided, radiating to his left shoulder, 8/10 in intensity, and associated with diaphoresis. He presented to sick call, where he was given aspirin 324 milligrams (mg) and transferred to the ED. On evaluation in the ED, his pain had resolved.

The patient was stable, afebrile, with a heart rate of 88 beats per minute, respiratory rate of 16 breaths per minute, blood pressure 105/70 millimeters of mercury, and pulse oximeter oxygen saturation of 97% on room air. His physical exam was significant for an irregularly irregular cardiac rhythm with no murmurs, rubs, or gallops appreciated. Labs were significant for a negative troponin performed at sick call, a repeat negative troponin five hours later in the ED, negative D-dimer, unremarkable complete blood count, and unremarkable electrolytes. The patient’s ECG was significant for atrial fibrillation with a ventricular rate of 72, normal axis, unremarkable intervals, and no ischemic changes, all consistent with his prior ECGs. Chest radiograph was without acute cardiopulmonary process.

Bedside POCUS TTE was performed with a PX ultrasound system (Fujifilm Sonosite, Inc, Bothell, WA) using the cardiac phased array 5-1 MHz transducer and cardiac settings. The patient was in atrial fibrillation at the time of the TTE. The scan was significant for normal EF, no wall motion abnormality, no pericardial effusion or tamponade, and fluttering motion of the mitral valve thought to represent the effect of regurgitant flow from an incompetent aortic valve (Videos 1, 2).

M-mode in the parasternal long-axis view to assess EPSS demonstrated a sawtooth pattern of anterior mitral valve fluttering (Image). The apical four-chamber view was significant for aortic regurgitation.

Given the concern that the sawtooth pattern of anterior mitral valve pattern represented a new-onset valvular pathology, including severe aortic regurgitation, the patient was admitted to the hospital for further workup. The next day, he underwent TTE performed by a radiology technician. He was again in atrial fibrillation. The cardiologist’s report largely affirmed the findings of the POCUS TTE. That said, although M-mode EPSS was performed, it did not detect anterior mitral valve leaflet fluttering, and the patient’s aortic regurgitation was quantified as “trivial.” The patient also underwent a myocardial perfusion study that was negative for ischemia or infarction. He was discharged with a cardiology referral and primary care follow-up.

**CPC-EM Capsule**

What do we already know about this clinical entity?

*Anterior mitral leaflet flutter on M-mode is known to occur with aortic regurgitation but is underrecognized as a sign of atrial fibrillation*.

What makes this presentation of disease reportable?

*It’s the first peer-reviewed emergency medicine case linking anterior mitral leaflet flutter on M-mode to atrial fibrillation*.

What is the major learning point?

*Mitral leaflet flutter on M-mode during E-point septal separation may indicate atrial fibrillation, even without significant aortic regurgitation*.

How might this improve emergency medicine practice?

*It enhances rapid bedside identification of atrial fibrillation using point-of-care ultrasound, aiding diagnosis and patient disposition*.

At his follow-up appointment with cardiology three weeks later, the patient reported that he did not feel that he had converted to sinus rhythm since his admission. He continued to remain symptomatic, with chest pain and dyspnea (for which he had been evaluated again in the ED the day after his discharge from the hospital) and was referred to electrophysiology for ablation.

## DISCUSSION

With the advent of 2D imaging and Doppler techniques, present-day echocardiography relies less on M-mode.^1^, ^2^ The peer-reviewed literature on the M-mode appearance of the mitral valve dates largely from the 1980s and early 1990s.^5^, ^6^, ^7^, ^8^, ^9^ This literature established that aortic regurgitation can cause anterior mitral valve leaflet fluttering detectable on M-mode.^1^, ^2^, ^7^, ^9^ This anterior mitral valve fluttering has been a proposed etiology of the Austin Flint murmur,^10^, ^11^ but at least one study reported no difference in the frequency of anterior mitral valve fluttering in patients with and without the Austin Flint murmur.^12^ These decades-old studies do not discuss POCUS. In the ED, POCUS TTE is commonly performed for patients presenting with chest pain, dyspnea, dysrhythmia, and palpitations. With the less frequent use of Doppler on TTE in the ED, M-mode EPSS may be used to quantify EF and support EF estimation based on visual assessment.^12^, ^13^

In the present case, the anterior mitral leaflet flutter observed on M-mode echocardiography was interpreted as a sequela of the patient’s incompetent aortic valve, consistent with the echocardiography literature. That said, subsequent TTE performed by the radiology technician and read by the cardiologist found only “trivial” aortic regurgitation. “Severe” aortic regurgitation is typically required to produce the mitral valve flutter.^1^, ^7^, ^9^

The cardiologist’s report did not provide an obvious alternative explanation for the M-mode anterior mitral valve flutter. Our examination of the peer-reviewed literature was likewise unrevealing. But similar M-mode tracings of anterior mitral valve flutter were located on non-peer-reviewed online sources, and these identified the finding as also indicative of atrial fibrillation.^3^, ^4^ As our patient was in atrial fibrillation at the time of the POCUS TTE, it seemed reasonable to attribute the observed anterior mitral leaflet flutter to a function of his atrial fibrillation.

Recognition of the anterior mitral valve flutter on M-mode echocardiography as a manifestation of atrial fibrillation is valuable for emergency physicians performing POCUS TTE on an undifferentiated patient with chest pain, dyspnea, palpitations, or dysrhythmia. In the case at hand, the patient was admitted with concerns for new-onset mitral valve pathology or aortic regurgitation. Had the consistency of the M-mode finding with atrial fibrillation been known to the emergency physicians, the interpretation of the POCUS TTE would have supported a diagnosis of symptomatic atrial fibrillation, which was the patient’s ultimate diagnosis on discharge from the hospital.

## CONCLUSION

Anterior mitral leaflet fluttering on M-mode echocardiography is consistent with atrial fibrillation in the appropriate clinical context. This association does not appear in the EM literature, and the frequency and quality of atrial fibrillation associated with this aberrant mitral leaflet flutter is poorly described in the available literature. This information is of particular relevance to emergency physicians assessing E-point septal separation as part of point-of-care ultrasound transthoracic echocardiography on the undifferentiated ED patient with chest pain, dyspnea, palpitations, or dysrhythmia. Where a patient is in atrial fibrillation and lacks severe aortic regurgitation, anterior mitral leaflet fluttering on M-mode may confirm symptomatic atrial fibrillation in a patient presenting with chest pain.

## Supplementary Information

Video 1 The anterior mitral valve is fluttering in the parasternal long axis (see arrow and label).

Video 2 The anterior mitral valve is fluttering in the parasternal short axis (see arrow and label).

## Figures and Tables

**Image:**
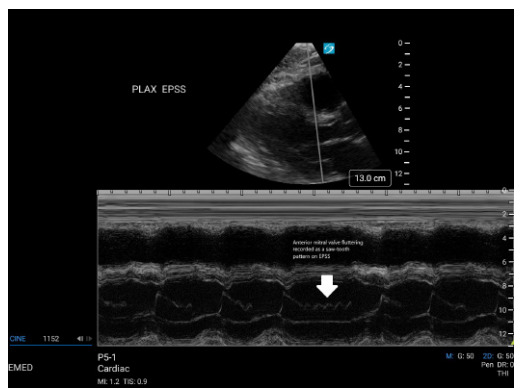
M-mode visualization of anterior mitral valve fluttering in parasternal long axis shows a sawtooth pattern (arrow). *PLAX EPSS*, E-point septal separation measured in the parasternal long-axis view.
